# Hybrid Immunity against SARS-CoV-2 Variants: A Narrative Review of the Literature

**DOI:** 10.3390/vaccines12091051

**Published:** 2024-09-14

**Authors:** Panagiota Tsagkli, Maria Geropeppa, Ioanna Papadatou, Vana Spoulou

**Affiliations:** Immunobiology and Vaccinology Research Laboratory and Infectious Diseases Department “MAKKA”, First Department of Paediatrics, “Aghia Sophia” Children’s Hospital, Athens Medical School, 11527 Athens, Greece

**Keywords:** hybrid immunity, COVID-19, SARS-CoV-2 variants, SARS-CoV-2 vaccination, COVID-19 immunology

## Abstract

The emergence of SARS-CoV-2 led to a global health crisis and the burden of the disease continues to persist. The rapid development and emergency authorization of various vaccines, including mRNA-based vaccines, played a pivotal role in mitigating severe illness and mortality. However, rapid viral mutations, leading to several variants of concern, challenged vaccine effectiveness, particularly concerning immune evasion. Research on immunity, both from natural infection and vaccination, revealed that while neutralizing antibodies provide protection against infection, their effect is short-lived. The primary defense against severe COVID-19 is derived from the cellular immune response. Hybrid immunity, developed from a combination of natural infection and vaccination, offers enhanced protection, with convalescent vaccinated individuals showing significantly higher levels of neutralizing antibodies. As SARS-CoV-2 continues to evolve, understanding the durability and breadth of hybrid immunity becomes crucial. This narrative review examines the latest data on humoral and cellular immunity from both natural infection and vaccination, discussing how hybrid immunity could inform and optimize future vaccination strategies in the ongoing battle against COVID-19 and in fear of a new pandemic.

## 1. Introduction

Since the emergence of SARS-CoV-2, it is estimated that more than 770 million individuals have been infected with the virus and almost 7 million have died, as of June 2024 [1]. The emergency authorization of mRNA vaccines was undoubtedly a valuable measure against severe disease and death during the pandemic in the previous two years. As of June 2024, thirteen different vaccines have been granted emergency use authorization by the World Health Organization (WHO) [2]. Among them, different technologies have been used: vaccines using protein subunits of the virus, such as the spike (S) protein (NVX-CoV2373 vaccine), and mRNA-based vaccines (BNT162b2), those containing non-replicating viral vectors (AZD1222—withdrawn in May 2024), and the inactivated ones (BBIBP-CorV) [3]. All of the aforementioned vaccine platforms contain antigens from the ancestral strain of the virus that reigned initially in the pandemic. However, rapid mutations of the virus have led to the appearance of new variants such as Beta, Delta, and Omicron that raised concern about immune evasion and posed a challenge for vaccine effectiveness [4].

Intense research on the immunity provided via natural infection or immunization was conducted with unprecedented velocity, offering the opportunity to shed light on several immunological aspects of this novel virus. Soon it became clear that although neutralizing antibodies—NAbs—offer protection against infection, their durability is short-lived and the main correlation for protection against severe disease and death derives from the cellular compartment of the adaptive immunity [5]. More specifically, CD8^+^ cytotoxic T-cell responses have been found to be an essential contributor to the protection against severe coronavirus disease–COVID-19—by clearing infected cells, especially in the setting of minimal antibody responses [6], while CD4^+^ helper T-cells are important for protective antibody responses holding an assistive role for CD8^+^ T-cells’ maturation and proliferation [7].

In this context, hybrid immunity is defined as a form of promoted immunity for convalescents who have been vaccinated against SARS-CoV-2. This term encloses vaccinated individuals who had one or more SARS-CoV-2 infections either prior or post immunization that have had a primary series of vaccination or a booster dose. Evidence shows that convalescent vaccinated people have on average 50-fold higher NAbs than convalescents who have not been vaccinated [8]. In light of the above, hybrid immunity derives from the two branches of adaptive immunity: the one provided via natural infection and the artificial one that vaccination can offer. As the landscape of SARS-CoV-2 epidemiology is constantly shifting with the emergence of new variants and breakthrough infections occurring in an increasing number of vaccinated individuals, hybrid immunity has become an established type of anti-SARS-CoV-2 immunity. So, it is imperative to elucidate the durability of the protection offered by hybrid immunity and the protective coverage across new variants.

In this narrative review, we will briefly summarize all the new data on the humoral and cellular immunity induced by SARS-CoV-2 natural infection and vaccination, as well as the advantages probably offered in the setting of hybrid immunity. We will primarily concentrate on research related to immunological memory generated by mRNA vaccines, as these vaccines constitute the majority of doses administered to date. We will also discuss based on this accrued evidence how current vaccination strategies could be optimized.

## 2. Immune Response to SARS-CoV-2 Infection

The role of antibodies for the inhibition of infection from SARS-CoV-2 and controlling the disease course is crucial. Therefore, the durability of antibody responses to SARS-CoV-2 is a well-investigated topic. Antibody responses can be categorized into short-lived and long-lived; the former originates from activated B-cells, which differentiate into short-lived plasma cells after encountering SARS-CoV-2 antigens and aim to mitigate viral replication. Durable antibody responses occur from long-lived plasma cells, residing in the bone marrow. These plasma cells have been generated in the germinal center reactions following infection and are responsible for producing large amounts of antibodies longitudinally [9].

The initial step in the immune response to SARS-CoV-2 infection involves innate immunity, with the recognition of the virus by specific cellular receptors that detect viral proteins and RNA, leading to the activation of signaling pathways that produce pro-inflammatory cytokines and type I interferons. The immune response involves antigen presentation to T-cells, which helps activate both CD8^+^ T-cells to kill infected cells and CD4^+^ T-cells to support B-cell antibody production.

Shortly after infection, circulating SARS-CoV-2-specific antibodies and neutralizing antibodies are detected in the majority of infected individuals [10]. NAbs seem to decrease in the first months following infection [11] and then achieve a plateau phase at 4–6 months after infection, remaining detectable in more than 80% of convalescent individuals 1 year after infection [12,13]. S-specific and RBD-specific antibodies display similar kinetics to NAbs and seem to remain detectable in the first year following infection, though in lower titers than shortly after infection [12,14,15].

Memory B-cells (MBCs) are a major component of immunological memory as they have a double-faceted function; upon an antigenic re-challenge, MBCs can rapidly differentiate into antibody secreting cells for an early burst of high-affinity antibodies, while they can also re-seed the germinal center reactions for the replenishment of the MBC pool [16]. Regarding SARS-CoV-2 infection, SARS-CoV-2-specific MBCs are detected within two weeks from infection [12,17]. MBC frequencies significantly increase in 3–6 months following infection, suggesting ongoing germinal center reactions [18] that are thereafter sustained for over 1 year [19].

Cellular immunity is an essential arm of immune response to infections. CD3 T-cells are subdivided into two major cell types, CD4^+^ helper T-cells and CD8^+^ cytotoxic T-cells. The early generation of SARS-CoV-2-specific CD8^+^ T-cells can be detected even within 7 days from the onset of symptoms with a typical peak at 14 days after the symptom onset [20]. SARS-CoV-2-specific CD8^+^ T-cells can be detected in up to 70% of convalescent individuals 1 month after the confirmed diagnosis and decline to 50% in the following 8 months [12,13]. However, with the delayed t_1/2_ of more than 190 days reported in the studies and the detection of memory SARS-CoV-1-specific CD8^+^ T-cells 17 years after infection, it can be postulated that SARS-CoV-2 infection induces long-lasting memory CD8^+^ T-cells [12,13,21]. Memory CD8^+^ T-cells tend to display a phenotype of effector memory and CD45RA^+^ effector memory [12,22]. Of note, severe disease and hospitalization have been associated with lower frequencies of SARS-CoV-2-specific total and memory CD8^+^ T-cells, in contrast to the correlation observed between disease severity, S-specific antibody titers, and S-specific memory B-cells [23,24,25].

Similarly, SARS-CoV-2-specific CD4^+^ T-cells can be detected shortly after infection and remain detectable in up to 90% of SARS-CoV-2-infected individuals in the following months of convalescence [12,13,26]. The longevity of SARS-CoV-2-specific memory CD4^+^ T-cells is supported by studies demonstrating an increasing t_1/2_ of 200 to 307 days [12,27]. In contrast to memory CD8^+^ T-cells, the majority of memory CD4^+^ T-cells display a central memory phenotype, followed by an effector memory phenotype [12,13]. Infection-induced SARS-CoV-2-specific memory CD4^+^ T-cells are predominantly T-helper 1 (Th1) cells, T-follicular helper (Tfh) cells, and CD4-cytotoxic (CTL) cells [27]. A heterogeneity in SARS-CoV-2-specific CD4^+^ T-cell responses has been observed, concomitantly to the reported heterogeneity in clinical outcomes of the infection [12], as CD4^+^ T-cell frequencies are influenced by older age and comorbidities [28], while gender has no effect on CD4^+^ T-cell responses [12,13]. Nevertheless, in contrast to CD8^+^ T-cells, which have been inversely correlated with disease severity, asymptomatic infection may induce lower frequencies of SARS-CoV-2-specific CD4^+^ T-cells compared to mild disease [21,29]. Systems biology methods have offered a massive contribution in unraveling the differences among mild and severe disease [30]. Multiple transcriptomic analyses underscore the significance of the type I interferon pathway in the pathogenesis of COVID-19, as interferon type I plays a key role in the regulation of T-cell differentiation [31].

## 3. Immune Response to Vaccination against SARS-CoV-2

Generally, the immune response to vaccination derives from the cooperation of the innate with the adaptive immunity, expressed initially as an interplay between antigen presenting cells (APCs) and naïve T-cells [32]. Novel vaccines against SARS-CoV-2 contain lipid nanoparticles that encapsulate mRNA, which encodes the SARS-CoV-2 S-protein. mRNA gains access into dendritic cells (DCs) at the site of the injection and as a result, S-protein is produced. DCs expose peptides on their surface, within major histocompatibility complexes (MHCs)—known as HLA in humans—and they move to the adjacent lymph nodes, where DCs are connected with the T-cell receptors of naïve T-cells [14,33]. With the priming offered by several secreted cytokines and chemokines such as the type I interferon, naïve T-cells are differentiated into effector cells with two main subtypes: cytotoxic T-cells and helper T-cells [34]. High-affinity S-protein antibodies are produced after the interaction of Tfh cells with antigen-specific B-cells [35]. The eventual purpose of an effective vaccine is to develop long-lived immunological protection.

The first encounter with a pathogen should be ‘remembered’, which leads to robust memory responses that either prevent re-infection or significantly reduce the severity of disease [36]. So, the main factor that predicts the protection provided by vaccination is immunological memory. Four compartments consist of the adaptive immunity offered via immunization: memory B-cells, memory CD4^+^ T-cells, memory CD8^+^ T-cells, and circulating antibodies [37]. Previous infection, vaccination, or hybrid immunity offer immunological memory with different and unique characteristics. Here, we will mainly focus on studies about immunological memory induced after mRNA vaccines as the vast majority of vaccination doses offered have been based on this technology to the present.

### 3.1. Memory T-Cells to Vaccination

Memory T-cells and specifically CD8^+^ T-cells play a key role in adaptive immunity. However, difficulties regarding measurement techniques and the absence of correlates of CD8^+^ T-cell-mediated protection make the deciphering of their role challenging. Generally, the immune response to vaccination derives from the cooperation of the innate immunity with antigen presenting cells (APCs) and the adaptive one with naïve T-cells [38]. It is now widely accepted that S-specific CD8^+^ T-cells are found in most individuals vaccinated with a two-dose scheme of primary vaccination with mRNA vaccines [39,40]. More specifically, Painter et al. found that CD8^+^ T-cells develop gradually after vaccination with low numbers after the first dose [41]. However, they increase after the second dose and thus 88% of the examined individuals demonstrate detectable CD8^+^ T-cells [41]. CD8^+^ T-cells develop before the antibodies and as a result, they initially play a significant role in the orchestration of the offered protection. It is also indicated that the pool of CD8^+^ T-cells is preserved in almost one third to half of the vaccinees even 6 months post-vaccination [42]. As for the specific phenotype of the detectable CD8^+^ T-cells, this is T-cell effector memory (CD8^+^ Temra CD45RA^+^ CCR7-), a type that resides in peripheral lymphoid organs [43]. Effector memory T-cells express cytokines such as IFN-γ and thus they have the capacity to proliferate [40]. With the emergence of new variants of concern (VOCs) such as Omicron (B.1.1.529, BA.2.86, XBB.1.5, EG.5.1, HV.1, and JN.1) [5] and the waning of humoral immunity, the need for vaccination with booster doses especially in older and high-risk populations has arisen. Memory CD8^+^ T-cell response—after mRNA vaccination—targets epitopes that are covered in all existing VOCs by now [40]. Accumulating data show that vaccinees primed by the ancestral strain of S-protein develop a T-cell response that cross-recognizes the current variants of concern [44]. A robust activation and proliferation of spike-specific CD8^+^ T-cells was observed post the third and fourth dose of mRNA vaccines; however, this differentiation was contracted after 1–2 months, with T-cells following a similar trajectory to after the second dose [45]. The recall ability, expansion of antigen-specific CD8^+^, and production of cytokines such as IFNγ and TNF remained covered prior to and post the third vaccination [45].

It is commonplace that memory CD4^+^ T-cells—contrary to memory CD8^+^ T-cells—are found in almost all of the vaccinees after a two-dose scheme vaccination [42,46]. S-specific CD4^+^ T-cell responses to mRNA vaccines are observed approximately 1 month after the second dose. The distinct role of CD4^+^ T-cells is the assistance of B-cells for antibody responses. After vaccination, there is a skew towards the Th1 subset that produces IFN-γ, TNF, and IL-2 [47]. Vaccinated individuals with an extended interval between the two doses of mRNA vaccines produce more IL-2-expressing memory CD4^+^ T-cells [48]. Almost 25% of the memory CD4^+^ T-cells generated after mRNA vaccines consist of Tfh cells [46]. Tfh cells are a special subpopulation of CD4^+^ T-cells necessary for B-cell differentiation into plasma cells and memory B-cells in the germinal centers of secondary lymphoid organs [49]. As a consequence, the quantity of Tfh cells is correlated with the magnitude of neutralizing antibodies’ response [41,46,50]. Tfh cells that reside in germinal centers are found in the lymph nodes of vaccinees for more than 6 months [51]. The durability of memory CD4^+^ T-cells generally is estimated to surpass 6 months post-vaccination [42,46]. They expand after additional doses and they are capable of cross-recognizing several spike epitopes covered in different variants [43].

Regarding other vaccine platforms, AZD1222 has also been shown to induce IFN-γ responses in clinical trials after one AZD1222 vaccine dose, without significant enrichment after a booster dose [52]; in real-world settings, IFN-γ responses to S1 and S2 proteins have been detected one month following a single AZD1222 vaccine dose [53]. The Sinopharm vaccine (BBIBP-CorV, an inactivated-virus vaccine combined with an alum adjuvant) was demonstrated to induce CD4^+^ and CD8^+^ T-cell responses after the first vaccine dose, with the second dose significantly enhancing CD4^+^ but not CD8^+^ T-cells [54]. Furthermore, compared to other inactivated vaccines, the study concluded that vaccinated individuals with BBIBP-CorV displayed lower T-cell response after one vaccine dose, but it was demonstrably induced after two vaccine doses [54]. Clinical trial data on a recombinant subunit vaccine manufactured by Novavax (NVX-CoV2373) supported the induction of a CD4^+^ T-cell response, skewed towards the Th1 phenotype [55]. Accordingly, in a study comparing NVX-CoV2373 to BNT162b2 vaccine-induced immunity, an induction of S-specific CD4^+^ T-cells was detected in the majority of participants vaccinated with NVX-CoV2373 (82%), albeit lower than BNT162b2 vaccines, while S-specific CD8^+^ T-cells were only detectable in 14% of NVX-CoV2373-vaccinated participants [56]. Furthermore, CD4^+^ and CD8^+^ NVX-CoV2373-induced T-cells recognized various VOCs from Alpha to Omicron.

### 3.2. Memory B-Cells and Antibodies to Vaccination

Two doses of mRNA vaccines elicit class-switched and affinity-matured memory B-cells. More specifically, there is a surge in frequencies of S- and RBD-specific memory B-cells between 3 and 6 months post-vaccination [57]. However, there are data that qualitive characteristics of affinity maturation after a two-dose scheme are suboptimal compared to infection [57]. An explanation for that could be the short interval between the two doses that may affect the priming time needed for affinity maturation to happen. Indeed, the extension of dose intervals between immunizations significantly improved the titers of antibodies [48]. Longitudinal studies on the durability of B-cells and antibodies have shown that although antibodies diminish over time, this reduction stabilizes approximately between 6 and 9 months after the second dose of primary vaccination [58]. However, quality continued to improve for at least 9 months [58]. Regarding the S- and RBD-specific memory B-cells, they remained stable over time and almost 50% of them engaged to Alpha, Beta, Delta, and Omicron variants. A third dose of a wild-type vaccine had a booster effect on Omicron-binding memory B-cells, and a positive correlation with neutralizing antibodies was observed. Conversely, antibody titers prior to the third vaccination inversely correlated with the fold-change of an antibody increase, indicating that high levels of circulating antibodies probably dampen the further protection provided by frequent anamnestic doses [58].

It is now clear that two doses of mRNA vaccines induce high titers of NAbs in most individuals; however, as mentioned above, they decline rapidly. There is evidence by a large study by Israel et al. that there is a steep 16-fold decrease in RBD-IgG titers from their peak over a 7-month period of time [59]. S-specific long-lived plasma cells are detected in a big proportion of vaccinees even 6 months post immunization. However, taking into consideration the decline in antibodies, it is evident that either there is a low number of plasma cells or they are not durable enough [60]. The waning of antibody levels and the emergence of new variants led to the need for booster doses especially in high-risk groups. A bivalent mRNA vaccine, which includes the ancestral spike protein and the muted Omicron subvariant BA.5, received approval for clinical use in the fall of 2022 [61]. However, immunization with the bivalent vaccine did not show a significantly better induction of neutralizing antibody titers against BA.5 compared to boosting with the original monovalent vaccine [62]. Nonetheless, data show that they have high effectiveness against severe COVID-19 and death [63]. An updated mRNA Omicron XBB.1.5 monovalent booster was approved for use in the fall of 2023 [64]. Data show that boosting with the mRNA Omicron XBB.1.5 monovalent vaccine significantly increased NAb titers against XBB.1.5, as well as EG.5.1JD.1.1, and JN.1, suggesting that it could provide enhanced protection against these emerging variants too [65].

Data on B-cell responses to other vaccine platforms are available, yet are limited with regards to mRNA vaccines. Memory spike-specific and memory RBD-specific MBCs are elicited shortly after two AZD1222 doses, although at lower frequencies compared to mRNA vaccines [66]. BBIBP-CorV was demonstrated to induce durable B-cell ELISpot responses to S1 and S2 proteins 12 weeks following a two-dose immunization schedule, in spite of waning antibody titers [53]. Spike-specific and RBD-specific MBCs have also been detected following 3, 5, and 6 months after two NVX-CoV2373 doses, but the MBC response was attenuated compared to mRNA vaccines; in comparison, NVX-CoV2373 induced a higher frequency of classical (CD21^+^CD27^+^) MBCs than mRNA vaccines [67].

With regards to different vaccine platforms than mRNA, AZD1222 elicited a robust IgG-specific SARS-CoV-2 antibody response, peaking at 4 weeks following a single vaccine dose and remaining relatively stable at up to 11 weeks [68]. Moreover, IgG anti-RBD antibodies induced by one AZD1222 dose were cross-reactive against viral VOCs, including Beta and Delta variants [69]; vaccinated individuals with AZD1222 were protected against pneumonia from the Omicron variant, in spite of the variant’s ability to avoid neutralization [70]. BBIBP-CorV was found to induce IgGs and NAbs against RBD following two vaccine doses at a lower [54], yet satisfactory, seroconversion rate. A third vaccine dose at 2 or 5 months after a two-dose primary schedule was proven to not only compensate for the declining antibody titers [71], but also enrich NAbs against Alpha and Beta variants [72]. Regarding the NVX-CoV2373 vaccine, it was found to induce IgG antibodies and NAbs in clinical trials at higher levels than convalescent individuals [55]. Effectiveness of NVX-CoV2373 against multiple VOCs was estimated at above 90% in adults [73]. Furthermore, in a study evaluating NAbs against Omicron sub-lineages, two vaccine doses displayed low NAb titers against BA.1 and BA.4/BA.5 sub-lineages, though a third dose induced NAb titers at levels comparable to three mRNA vaccine doses [74].

## 4. The Phenomenon of Hybrid Immunity

With the highest proportion of the population worldwide having recovered from SARS-CoV-2, it is of great interest to decipher the various aspects of hybrid immunity. Data on the humoral response have shown that individuals with previous infection and two doses of mRNA vaccines develop high anti-S and anti-RBD IgG titers with a 7-month durability, comparable to the response induced by a two-dose vaccination scheme alone but significantly higher than that generated only from infection [75]. Moreover, Chen et al. found that prior SARS-CoV-2 infection led to a more even spike-specific recognition across emerging variants—including Omicron—compared to a two-dose mRNA vaccine. After vaccination, COVID-19 convalescents showed improved antibody responses, similar to those receiving a third vaccine dose. Additionally, convalescent individuals showed more gradual antibody decay and improved durability after vaccination, particularly those with cross-coronavirus-reactive anti-S2 IgG in their MBCs. This suggests that pre-existing B-cell memory to endemic coronaviruses may contribute to more stable and long-lasting anti-SARS-CoV-2 antibody responses [75]. Hybrid immunity is also effective when vaccination precedes infection, producing high titers and broad NAb responses across different variants. This immunity shows robust durability, with NAb titers remaining stable for 6 months and declining less than two-fold in most individuals. People with hybrid immunity maintain significantly higher NAb titers compared to those who were only vaccinated or only infected. Additionally, higher nasal IgG and IgA levels are observed in hybrid immunity individuals, providing prolonged protection up to 10 months post-vaccination [76]. In a large retrospective study, Goldberg et al. compared the incidence of SARS-CoV-2 infection over a certain period of time in cohorts with different histories of immunity-conferring events including infection and vaccination [77]. Investigators observed that although the protection against re-infection had diminished over an 8-month period of time in all cohorts, this protection was higher among those that had recovered from previous infection and had also received a dose of vaccine prior to or post the infection [77]. These findings clearly underline the priming role of immunity induced by infection and the booster effect of immunization.

**Figure 1:**
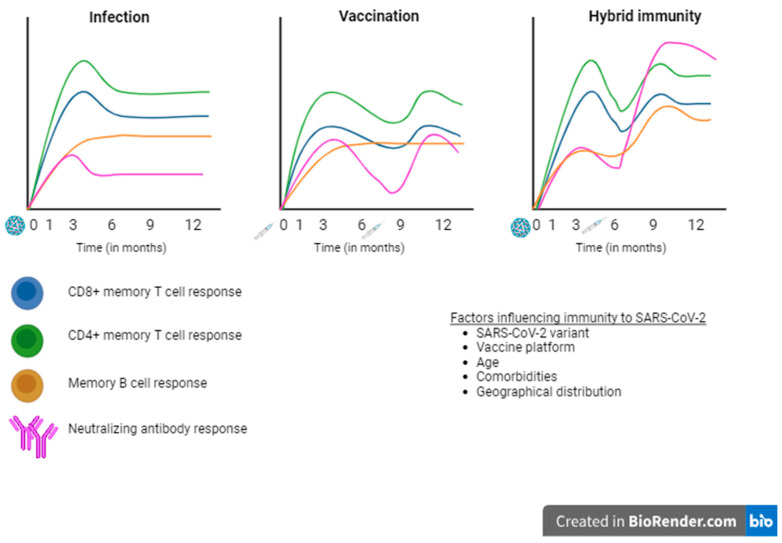
Kinetics of the various aspects of immune response to SARS-CoV-2 acquired by natural infection, vaccination, and hybrid immunity. Adapted from [80].

Studies on humoral response have demonstrated that individuals with prior infections maintain higher levels of RBD-specific MBCs, as well as RBD-specific IgG and IgA, compared to those without prior infection, 3 and 6 months after receiving a two-dose vaccination [78]. Interestingly, these differences in humoral response between the two groups recovered after a third dose. Bowman et al. implemented a systems serology approach to decode the qualitive differences in humoral response to vaccination between previously infected and naïve groups. They found qualitatively superior antibody responses in individuals with hybrid immunity that was associated with increased Fc-receptor-binding antibody levels. These antibodies may promote the viral clearance and improve the ability to engage the S2 epitopes on the S-protein, which is more conserved across the variants, but this epitope is probably not available in mRNA vaccines that bear the pre-fusion-stabilized S-protein where S1 is immunodominant. Assessment of the cellular immunity brought to light a distinct subpopulation of IFN-γ- and IL-10-expressing memory SARS-CoV-2 S-specific CD4^+^ T-cells that are formed only in the previously infected group and in the naïve group. This unique cytokine profile could not be generated by the naïve individuals after additional doses of vaccination [78,79]. The differences between infection-, vaccination-, and hybrid immunity-acquired SARS-CoV-2-specific immunity are displayed in Figure 1.

With the ongoing emergence of VOCs altering the landscape, it is a challenge for researchers to elucidate the breadth of the protection that hybrid immunity provides. The questions that should be answered so that we can evaluate the protection offered against several variants involve whether infection occurred before or after vaccination and which type of variant caused the infection. In more detail, there are data showing that hybrid immunity induced by triple vaccination and heterogenous infections by VOCs such as the ancestral strain, Alpha, and Delta afforded a cross-reactive immunity against pre-Omicron variants but a diminished protection against Omicron [81]. However, a study on cellular immunity has shown that former SARS-CoV-2 infection plus vaccination with the BNT162b2 mRNA vaccine elicited polyfunctional memory T-cells capable of responding to several VOCs including Omicron, despite the fact that neutralizing antibodies could not neutralize them [82]. Breakthrough Omicron infection in triple-vaccinated individuals only moderately primes immune response to Omicron sub-lineages but increases cross-reactive protection against other VOCs [81]. Conversely, Malato et al. supported that breakthrough BA.1 or BA.2 infection in vaccinated persons afforded higher protection against the BA.5 subvariant than infection with pre-Omicron variants; however, they underline that BA.1 or BA.2 infections occurred closer to the period of BA.5 dominance than infections with previous variants [83]. Interestingly, an initial infection with the wild-type variant followed by vaccination and then an infection by Omicron (BA.1) may increase the possibility for Omicron re-infection, as Omicron neutralizing antibodies are decreased [81]. The fact that immune boosting offered by Omicron infection seems to be affected by the type of previous SARS-CoV-2 exposure triggered hypotheses about the phenomenon of immune imprinting and may explain the multiple re-infections observed with Omicron variants. Park et al. accordingly found that breakthrough infection with the antigenically shifted Omicron variant mainly recalled memory B-cells targeting the epitopes that are common in the previous SARS-CoV-2 strains rather than stimulating naïve B-cells programmed to recognize Omicron S-specific epitopes [82]. Nonetheless, real-world data on the protection of hybrid immunity against symptomatic Omicron infection by a large nationwide study in Qatar showed that the effectiveness of previous infection plus two doses of BNT162b2 against BA.2 infection was 55.1% (95% CI, 50.9 to 58.9), compared to that of previous infection plus three doses of BNT162b2 being 77.3% (95% CI, 72.4 to 81.4) [84]. These percentages were higher than the effectiveness provided by infection or vaccination alone. Moreover, all conferring events offered robust effectiveness of more than 70% against severe or fatal COVID-19 due to BA.2 infection. However, accumulating evidence indicates that the protection offered is short-lived and re-infection can occur as few as 3 months after previous COVID-19 [85]. A Danish study found that a percentage of participants were re-infected with BA.2 only 20 days after a BA.1 infection [86].

Regarding the inactivated vaccine, a study from Guo, Li et al. in China including participants that were infected and then vaccinated showed that vaccination increased the titers of prototype spike-specific IgG, RBD-specific IgG, and nucleoprotein-specific IgG [87]. Similar findings also applied for infected–vaccinated participants before and after vaccination for Delta spike-specific IgG and BA.1 spike-specific IgG. Further evaluations of plasma samples from infected–vaccinated participants at 6 months, 1 year, and 2 years after infection suggest that inactivated vaccines increased neutralizing antibody titers. Interestingly, neutralizing antibody titers did not differ between participants who received one dose or two doses of the vaccine. However, in recovered–unvaccinated participants at the 2-year follow-up, neutralizing antibody ID50 titers were significantly lower for BA.1, BA.1.1, BA.2, BA.4/5, BF.7, BQ.1, and XBB than the ancestral strain. According to the same study, in unvaccinated individuals who recovered from COVID-19, SARS-CoV-2-specific memory B-cell responses to the S-protein and RBD were detectable two years post-infection, regardless of illness severity. MBCs were cross-reactive between the prototype virus and variants like Delta and BA.1, indicating that SARS-CoV-2 variants do not completely escape the initial immune response to the ancestral strain. Infected individuals who were also vaccinated had higher frequencies of ancestral S-protein and RBD (*p* = 0.014), Delta RBD, and BA.1 RBD MBCs compared to unvaccinated individuals. The number of MBCs did not significantly differ between participants who received one or two vaccine doses. Ex vivo ELISpot assays revealed no significant differences in IFNγ T-cell responses to spike and nucleoprotein peptide pools in both infected–unvaccinated participants 1–2 years post-infection and infected–vaccinated participants 5 months after vaccination. Similarly, there were no significant differences in cytokine production (IL-2, IFNγ, TNF-α) by CD4^+^ and CD8^+^ T-cells between spike and nucleoprotein pools, or between ancestral and BA.1 strains. Vaccination did not significantly alter these T-cell responses, regardless of the number of vaccine doses. These findings indicate that, similar to other variants, mutations in Omicron likely do not impair T-cell responses generated by natural infection or vaccination, likely because T-cell epitopes remain largely conserved.

Homologous vaccination with products of the same technology is the main approach in many countries; however, administering heterologous vaccination seems to be an advantageous alternative. An original study by Zou et al. has demonstrated that the anti-RBD IgG levels and the number of RBD-specific MBCs were similar in individuals who had been vaccinated with two doses of an inactivated vaccine (BBIBP-CorV or CoronaVac) and a booster dose of an mRNA vaccine (BNT162b2 or mRNA 127) to those who had received three doses of an mRNA vaccine or to convalescent patients who had been followingly vaccinated with an mRNA vaccine [88]. Moreover, the numbers of S1-specific IL-2 and IFN-γ T-cells were greater in the group vaccinated with the heterologous scheme. Noteworthily, cross-binding activity of the antibodies was observed against Beta, Delta, and Omicron variants with the heterologous vaccination [88].

Accordingly, Ho at al. found firstly that individuals with hybrid immunity generated a more robust neutralizing activity than their counterparts that were vaccinated without a history of a previous infection and secondly that heterologous vaccination with an adenovirus vector vaccine (AD5-nCOV) and an mRNA vaccine produced higher levels of NAbs compared to a single-scheme immunization both in males and females [89]. This is a very interesting finding since viral vector vaccines are widely used in Latin America and Asia.

## 5. Factors That Influence Immune Response to Vaccination against COVID-19

Heterogeneity in immunogenicity to vaccination among individuals is a common phenomenon. Inherent characteristics of the host contribute to differences in immune response [90]. Numerous studies show that the vaccine-induced humoral immunity varies by gender and age. Females generally develop stronger antibody responses than males, influenced by hormonal differences like estrogen boosting immune responses [91]. Age also impacts immunity, with older adults showing diminished responses due to immune senescence. For example, seropositivity for IgG anti-S titers post COVID-19 vaccination was higher in younger individuals and females across different vaccines like CoronaVac and BNT162b2 [92,93]. Furthermore, while two doses improve antibody levels in older adults, younger people still show a more robust immune response [94]. The findings highlight the importance of considering sex and age in vaccination strategies, especially for older adults.

An intrinsic factor that also seems to affect the response to infection or vaccination is the HLA system. A large-scale study by Xie et al. conducted in the United Kingdom confirmed that the HLA-DQB1:06 allele family is correlated with an enhanced anti-RBD IgG to initial COVID-19 vaccination and fewer breakthrough infections [95]. Other studies found that HLA variations, like DQA1*3:03, DRB1*3:01, and DRB1*7:01, enhance serological responses post-vaccination. Conversely, other studies reported reduced antibody responses in individuals with specific HLA variations, such as A*3:01 and B*35:01. Thus, the data are conflicting and more studies are needed to establish conclusions [96].

Vaccination provides significant benefits for individuals previously infected with COVID-19, though the required doses may vary. Previously infected individuals, especially healthcare workers, exhibit higher antibody levels after the first dose (of vaccines like CoronaVac and mRNA vaccines) compared to infection-naïve individuals [97]. For those previously infected, a second dose often does not further increase antibody levels, suggesting that their prior infection serves as an initial immunological boost. This pattern is consistent across various vaccine types, emphasizing the strong antibody response from just one dose in those with prior infection [97]. However, completing the vaccination cycle is still recommended for robust immunity.

Immunocompromised individuals, including cancer, dialysis, and transplant patients, often exhibit diminished immune responses to COVID-19 vaccines. Cancer patients show varied seroconversion rates, with many not achieving adequate antibody levels after two doses of mRNA vaccines [98,99]. Age, gender, smoking habits, and cancer type influence vaccine effectiveness, and a third dose can significantly boost antibody levels in those with low initial responses [100]. Dialysis patients generally achieve high seropositivity after the second dose, but their antibody levels decline rapidly, necessitating booster doses [101,102]. Factors like age and immunosuppression affect their response to vaccines, with mRNA-1273 showing better efficacy than other vaccines [103]. Transplant recipients, particularly those on immunosuppressive therapies, demonstrate lower seroconversion rates compared to healthy individuals [104,105]. The timing of vaccination post-transplant and the type of immunosuppressive treatment are critical factors influencing antibody production [104,106]. A three-dose scheme enhances immune responses in many transplant patients, although a significant portion remain seronegative [107]. Overall, additional strategies, such as booster doses and careful timing of vaccination relative to treatments, are essential to improve vaccine efficacy in these vulnerable populations.

## 6. Future Perspectives

Although the COVID-19 pandemic has come to an end and a shift to an endemic situation has now been established, the risk for severe disease remains, especially for high-risk groups [108]. In the absence of a correlation to protection, the evaluation of the offered benefit from vaccination is multifaceted. Particularly, in the context of the emergence of new variants, constant reassessment of vaccine effectiveness is required in order to optimize the existing vaccination formulations. It is widely accepted that vaccines offer a robust protection against severe disease and death; however, effectiveness against infection is low and short-lived [58]. Hybrid immunity seems to be an advantage, since it provides high breadth and durability of antibody response [109]. However, even this form of immunity wanes over time. Noteworthily, a hurdle to an overall protection is the phenomenon of imprinting, by which the activation of memory B-cells generated by primary immunization results in antibody responses mostly against the ancestral strain following booster doses [110]. Data indicate that cellular immunity does not seem to be restricted by prior vaccination. Studies on adults and children have demonstrated that breakthrough infection boosts vaccine-primed S-specific T-cell responses and produces a repertoire of T-cells that can recognize multiple SARS-CoV-2 epitopes [111]. Furthermore, prior vaccination does not inhibit the induction of multi-specific T-cell responses.

Improved versions of the existing vaccines that could elicit better humoral and cellular responses should be a high priority. There is a need for more antigenic targets except for S-variants to be explored. These epitopes could promote stronger cellular immunity responses. Recent research demonstrates that epitopes such as SARS-CoV-2 nucleoprotein and viral RNA polymerase NSP12 are really promising [13,112]. Moreover, enhancing the mucosal immunity could play a pivotal role in order to avoid infection and subsequent transmission especially of Omicron sub-lineages. Current intramuscular vaccines do not elicit a robust mucosal immunity at the upper and lower respiratory tract [113]. A recent study on macaques showed that intratracheal boosting with a bivalent Ad26-based SARS-CoV-2 vaccine resulted in the significant induction of mucosal humoral and cellular immunity and almost full protection against the SARS-CoV-2 BQ.1.1 variant [114]. This formulation could be used on humans by inhalation or nebulization. There are also hypotheses that boosting the innate immune responses—a process called trained immunity—would probably protect from re-infection and severe infection as well [115]. This process is antigen-independent and it is based on epigenetic and metabolic rewiring of innate cells. Vaccines such as Calmette–Guerin (BCG), measles–mumps–rubella (MMR), and influenza vaccines may offer heterologous protection [116], while trained immunity could possibly act complementarily to vaccination.

A significant body of studies highlights that longer intervals between infection and vaccination, up to 400 days, lead to the greatest improvements in antibody titers and better cross-neutralization, particularly against the BA.1 Omicron variant [48,117,118]. These data emphasize the importance of the interval between infection and vaccination in enhancing the neutralizing response, suggesting that the order of exposure (infection before or after vaccination) does not inherently affect antibody development. Furthermore, this study supports the idea that longer infection–vaccination intervals improve antibody responses, even with delayed vaccination.

It should also be taken under consideration that the field of ‘hybrid immunity’ is becoming more and more complex since different variants emerge, new vaccines are produced, and there is a wide diversity of individual exposure histories. Mathematical models incorporating various parameters such as epidemic history of a population (e.g., circulating strains and exposure order) could predict future epidemic activity and contribute to the optimization of the existing vaccination program [119]. Of note, SARS-CoV-2 transmission and COVID-19 distribution are heavily influenced by age-dependent factors, such as contact rates, susceptibility, and disease severity. Children, with high contact rates, play a significant role in viral spread but are less susceptible and less likely to develop severe disease. In contrast, susceptibility and severity increase with age, making older individuals and those with comorbidities more vulnerable, thus prioritizing them for vaccination. Vaccines aim to induce neutralizing antibodies and reduce infection, hospitalization, and mortality. Despite varying efficacy across vaccines and age groups, they generally offer substantial protection, though not complete, particularly against emerging variants. Vaccination strategies must be tailored to these factors [120].

A recent surge of SARS-CoV-2 cases during 2024 has underscored the unmet need for a better protection due to new variants of interest. The development of next-generation vaccines is of pivotal importance. The idea of a pan-coronavirus vaccine seems to be the most promising one [121]. The aforementioned approach targets either all β-coronaviruses or multiple variants of a certain species such as SARS-CoV-2.

Further research is still required in order to unravel the complexity of immunity offered by SARS-CoV-2. Remarkably, systems biology studies offer a valuable approach for analyzing vaccine-induced responses and identifying the cellular and molecular markers associated with immunogenicity. Transcriptomics may be used as a predictive biomarker for tracking gene signatures in vaccinated individuals, helping to identify responders and non-responders to immunization. This insight could eventually lead to the development of more effective, personalized vaccination schedules, particularly for protecting high-risk groups. In this way, the highest level of protection could be offered and public health will be secured.

## Data Availability

As this is a review article, no data were collected from patients.
